# Removal and Mechanism of Cadmium, Lead and Copper in Water by Functional Modification of Silkworm Excrement Biochar

**DOI:** 10.3390/polym14142889

**Published:** 2022-07-16

**Authors:** Pengyang Bian, Yixuan Liu, Xiaoqin Zheng, Weibo Shen

**Affiliations:** 1 College of Natural Resources and Environment, Northwest A&F University, Xianyang 712100, China; bianpy@nwafu.edu.cn (P.B.); liuyx20@nwafu.edu.cn (Y.L.); 2 Qinba Ecological Protection Center, Hanzhong 723000, China; zkyjys123@126.com; 3 Institute of Soil and Water Conservation, Northwest A&F University, Xianyang 712100, China

**Keywords:** silkworm excrement biochar, functional modification, heavy metals, competitive adsorption, adsorption mechanism

## Abstract

A new type of biochar, called GBC, was prepared from silkworm excrement, and then modified by chitosan combined with pyromellitic dianhydride. The removal of mono-metal and polymetals (Pb, Cd and Cu) from an aqueous solution by GBC was investigated in this research. Compared to unmodified biochar, the removal rate of Pb and Cd by GBC was about 12% higher, while that of Cu was about 94.6% higher. It also shows the types of functional groups in biochar have a great impact on their adsorption. The removal of Pb is mainly involved in the N-C=O functional group, the removal of Cd is mainly involved in N-containing functional group and C=C bond, and that of Cu is mainly involved in N-containing functional group, carboxyl group, hydroxyl group, and a carbonyl group. Five adsorption–desorption cycles of GBC were carried out, and it was found that the adsorption capacities of GBC for Pb, Cd and Cu decreased by 7.28%, 10.78% and 6.07%, respectively, indicating that GBC had a good renewable performance. The adsorption capacity of GBC for Cu in different water samples is between 89.62 and 93.47 mg·g^−1^, indicating that GBC has great application potential for the removal of Cu in wastewater.

## 1. Introduction

Due to industrial activities, wastewater containing heavy metals is frequently discharged into water bodies, resulting in many environmental problems [1]. Contamination of water and soils with heavy metals has become a global environmental problem due to their toxicity, carcinogenicity and adverse effects on ecosystem health and food security [2]. Once heavy metals such as cadmium (Cd), lead (Pb) and copper (Cu) enter the water, they will enter the human body with the food chain, thus endangering human health [3]. Because of this, the exploration of heavy metal pollution methods that are economic and effective has become a top priority to ensure environmental safety.

The most frequently used methods to remove heavy metals in water are the following: (i) chemical precipitation; (ii) adsorption, (iii) ion exchange; and (iv) ultrafiltration [4,5,6]. However, these methods may have high operating requirements and high operating costs, and they may also lead to new environmental problems associated with energy consumption [7]. Among these four methods, adsorption is considered to be economic and effective for removing heavy metals from wastewater, especially under medium or low concentrations of ions [8]. The key to this is to find an economical and efficient adsorbent.

Among many adsorbents, biochar and activated carbon are commonly used for pollutants in water. Biochar is cheaper and easier to make than the activated one [9]. While the price of commercial activated carbon is around USD 3000 per ton [10], some studies provide an estimation of production costs of biochar at USD 51–386 per ton [11]. Biochar production is cheaper than activated carbon, but biochar has a lower specific surface area and fewer functional groups. This means that the adsorption capacity for pollutants in an aqueous solution is usually limited [12]. This hinders the further application of biochar in environmental governance. Therefore, to improve biochar’s performance in absorbing heavy metals and other pollutants and to provide more adsorption sites, its modification would be very helpful.

The surface modification method is widely used due to its simple modification process and good adsorption effect. Biochar-based materials are usually obtained by impregnating or capping adsorbents on biochar surfaces, especially regarding adsorbents with unique physical and chemical properties and high adsorption capacity [13,14,15]. Examples include but are not limited to adsorbents containing carbonaceous structures such as sulfadiazine, amino, and naphthenic acid [16]. After surface modification, the number of adsorption sites, functional groups, or other specific adsorption structures on biochar will increase [17], which will improve biochar’s adsorption capacity in terms of heavy metals and other pollutants.

Chitosan is a low-cost natural polymer material, and its content in nature is second only to cellulose. The molecular chain of chitosan contains many active groups, such as amino and hydroxyl. These active groups can participate in alkylation, esterification, acylation, carboxylation, and other chemical reactions [18]. This characteristic makes chitosan potential to be applied to the surface functionalization of biochar. However, chitosan’s adsorption capacity is still insufficient, because its specific surface area and active sites are limited [19,20]. Pyromellitic dianhydride (PD) is an important raw material for manufacturing polyimide polymer materials, and its molecular structure has two anhydrides. The two anhydrides in the PD molecule would have reactions with the amino group in the chitosan structure to form (-CONH-), which makes it cross-linked on the chitosan. The remaining anhydrides not in a reaction can be hydrolyzed into carboxyl groups [21]. Amino, carboxyl, and other functional groups would interact strongly with heavy metals. Those interactions can include but are not limited to ion exchange, electrostatic attraction, and surface complexation [22,23].

For the above reasons, in this study, chitosan was used to modify the biochar to introduce amino functional groups. Then, through the reaction of amino and anhydride in the PD molecule, PD was introduced into the modified biochar. This not only provides amino functional groups for biochar but also oxygen-containing functional groups. These functional groups could increase biochar’s adsorption performance in terms of heavy metals in an aqueous solution.

The preparation of adsorbents from agricultural and forestry wastes is a feasible method for processing heavy metal polluted water, due to adsorbents’ wide source and low preparation cost [24,25]. At present, biochar made from straw, livestock manure, and other wastes has been widely used to remove heavy metals and other pollutants in water. However, biochar made from silkworm excrement (a typical agricultural waste) is rarely reported to be used as a way to remove those mentioned. China′s sericulture has a long history: as the birthplace and main producing area of silk, there are many silkworm breeding areas. The annual output of silkworm excrement can reach more than 1 million tons [26]. Hence, silkworm excrement, as raw material for biochar preparation, has a wide range of sources.

At present, the modification of biochar by chitosan combined with PD is rarely reported, and the adsorption mechanism of combined modified biochar for heavy metals is not clear. In this study, biochar was prepared from silkworm excrement, and then it was modified by chitosan and PD to remove Pb, Cd, and Cu in an aqueous solution. The purpose of this study is: (1) to characterize silkworm excrement biochar modified by chitosan and PD, and (2) to assess its adsorption performance and adsorption mechanism on heavy metals. Hopefully, this would shed some light on removing heavy metals in water and discover a new way to utilize agricultural waste.

## 2. Materials and Methods

### 2.1. Materials

Solutions that were used were made with deionized water (18.2 ΩΜ) (Nanopure water, Barnstead, NH, USA). All reagents are analytically pure. Pb(HNO_3_)_2_, (Cu(NO_3_)_2_ ·3H_2_O), (Cd(NO_3_)_2_·4H_2_O), chitosan, and PD were purchased from Aladdin (China, Shanghai). Silkworm excrement was purchased in Taoyuan Town, Suining County, Jiangsu Province. Before use, silkworm excrement was dried, and then it was filtered through a 0.149 mm sieve and then spared.

### 2.2. Preparation of Biochar

The silkworm excrement powder was placed in a tubular furnace and pyrolyzed in a nitrogen atmosphere at a heating rate of 5 °Cmin^−1^ for 2 h at 600 °C. The original biochar was marked as BC.

The modification method of chitosan of silkworm excrement biochar was improved based on the method of Zhou et al. [25]. Detailed operation steps were as the following: 1.0 g of chitosan and 1.0 g BC were added to 50 mL of 2% concentration acetic acid. The acetic acid solution including chitosan and BC was then stirred in a water bath at a temperature of 50 °C for 30 min. The solution was then added drop by drop to 300 mL NaOH solution with a concentration of 1%. It was then placed under room temperature for 24 h, and it was then filtered to obtain a preliminary biochar sample modified by chitosan. At this time, NaOH was washed out with ultrapure water, and then the chitosan-modified biochar samples were placed in the oven at 60 °C for 24 h. After that, the samples were filtered through a 0.149 mm sieve for further modification.

Then, PD was used to further modify the chitosan-modified silkworm excrement biochar, detailed steps are as the following: 1.5 g PD was put in 50 mL dimethylformamide solution and then the solution was stirred fully. Then, 0.5 g chitosan-modified silkworm excrement biochar was added into a dimethylformamide solution containing PD. The mixture was placed in a 50 °C water bath for 5 h and then it was cooled at room temperature. It was then rinsed with dimethylformamide solution and rinsed with ultrapure water after cooled down to room temperature. Then, it was rinsed with 1% NaOH solution, and finally, it was rinsed with ultrapure water to neutralize. The sample was then placed in an oven at a temperature of 60 °C for 24 h. This sample was labeled as GBC, which was made from silkworm excrement biochar modified by chitosan combined with pyromellitic dianhydride.

### 2.3. Characterization of Samples

The surface morphology of the sample was observed by scanning electron microscopy (JEOL JSM-6700, Tokyo, Japan). In addition, a Specific Surface Area and Micropore Analyzer was then used to measure the area (ASAP 2020, Micromeritics, Boston, MA, USA). A certain amount of dried sample was weighed and placed in the measuring elbow, and the adsorption and desorption of nitrogen were carried out in a liquid nitrogen environment. Finally, the sample’s specific surface area was measured according to Brunauer–Emmett–Teller (BET) method. In addition, the sample was degassed for 2 h before BET determination. The content of ash was determined through the way of heating the sample to 750 °C. The functional groups of the sample were characterized by Fourier transform infrared spectrometer (FTIR, Nicolet 5700, Tokyo, Japan). FTIR scan frequency is 32, wave number range is 400–4000 cm^−1^, and resolution is 4 cm^−1^. X-ray diffractometer (XRD, D8-Advance; Bruker AXS, Berlin, Germany) was applied to the analysis of the crystal composition of the sample surface. The step length of the XRD measurement was 0.02, and the scanning range was 5–85°. In addition, X-ray photoelectron spectroscopy (XPS, Krato AXIS Ultra DLD, Tokyo, Japan) was then applied to analyze the composition and the different categories of elements on the sample surface.

### 2.4. Absorption Experiments

Please refer to the Supplementary Materials for a detailed description of the adsorption experiments.

## 3. Results and Discussion

### 3.1. Characterization of Biochar

The SEM characterization results of BC and GBC are in Figure S1. It suggested the surface of GBC (Figure S1b) was rougher than that of BC (Figure S1a). In addition, many pore structures can be observed on the surface of GBC. The difference between BC and GBC surfaces indicated that after modification with chitosan combined with pyromellitic dianhydride, the morphology of silkworm excrement biochar had changed significantly.

XRD patterns provide information on the crystal structure and phase composition of biochar. The XRD results of BC and GBC are presented in Figure S2. It presents that multiple peaks can be detected in BC and GBC, indicating that there are a certain number of mineral crystals in BC and GBC. After modification, the peak of KCl (corresponding with GBC at 3) and the peak of CaCO_3_ (corresponding to GBC at 4) disappeared. This indicated that the phase composition on the surface of silkworm excrement biochar was changed after modification by chitosan combined with pyromellitic dianhydride.

The specific surface area and pore volume of BC and GBC are presented in Table S1. It presents that the specific surface areas of BC and GBC were 28.4 and 68.2 m^2^·g^−1^, respectively, and the pore volumes of BC and GBC were 0.031 and 0.087 cm^3^·g^−1^. The specific surface area and pore volume of GBC were more than twice that of BC. This indicates the specific surface area and pore volume of silkworm excrement biochar significantly increased after chitosan combined with pyromellitic dianhydride modification. In addition, the pore volume measurement results of BC and GBC were consistent with the morphology results observed by SEM, i.e., the surface of GBC contained many pore structures. The contents of ash in BC and GBC were 29.6% and 21.8%, respectively. After modification, the ash content of GBC decreased, which may be due to the lack of KCl and CaCO_3_ in the modified GBC.

Some studies have shown that the functional groups contained in biochar are highly relevant to its properties, both physically and chemically. These properties will affect biochar’s adsorption performance [23]. In this study, the functional groups of BC and GBC were examined by FTIR (their spectrum see Figure S3). Generally, the peaks at 3550~3200 cm^−1^ are relevant to the stretching vibration of the alcohol or phenolic hydroxyl (-OH) [22]. The peaks at 2990~2880 cm^−1^ are caused by C-H stretching vibrations in aliphatic methyl (-CH_3_) or methylene (-CH) groups [27]. The peak at 1600 cm^−1^ is possibly related to the blue shift at 1720 cm^−1^, which is due to the C=O stretching vibrations in carboxyl and carbonyl groups [28,29]. The shift of this peak may be related to the changes in protonation, atom proximity, steric hindrance, dielectric strength, hydrogen bond strength, or polarity [30]. The peaks at 1421 cm^−1^ and 873 cm^−1^ regarding BC are caused by the carbonate vibration [27]. For GBC, the peaks at 1386 cm^−1^ are due to the stretching vibration of COO- [31], which indicates that there are carboxyl groups in GBC. The peak at 1159 cm^−1^ is due to the stretching vibration of the O-H [32]. The peaks at 533 cm^−1^ and 461 cm^−1^ are from the existence of aluminum salts [29]. The peaks in the 1150~1000 cm^−1^ range are from the C-O stretching vibrations in the polysaccharides [29]. The peak at 796 cm^−1^ is caused by the out-of-plane bending vibration of C-H in aliphatic and aromatic compounds [33]. After modification, the peak of BC at 1421 cm^−1^ disappears, while for GBC, peaks caused by COO- vibration and O-H vibration appear at 1386 cm^−1^ and 1159 cm^−1^ separately. This indicates that new oxygen-containing functional groups are added to its surface after it is modified by chitosan combined with pyromellitic dianhydride.

The elemental composition of biochar was determined by XPS. The XPS spectra of BC and GBC are in Figure S4. Figure S4a is the XPS full spectrum scan of BC, and Figure S4b is the XPS full spectrum scan of GBC. It can be seen from Figure S4b that the peaks of O1s corresponding to GBC at 1 and N1s corresponding to GBC at 2 are significantly stronger than those corresponding to BC at 1 and 2. The C, N, and O contents of BC and GBC determined by XPS are shown in Table S2. It can be seen from Table S2 that the C content of BC and GBC was 73.81% and 61.23%, the N content was 2.31% and 9.27%, and the O content was 22.63% and 28.04% separately. This result showed that after modification, many N-containing and O-containing groups appeared in GBC.

XPS of C1s and N1s of BC and GBC are shown in Figure S5. The N1s element of BC has a peak near 400.0 eV (Figure S5b). The N1s of GBC can be decomposed into three peaks (Figure S5d), which are composed of N-H (399.2 eV), N-C=O (399.8 eV), and N-C (400.6 eV) [34,35]. The C1s peak of BC is decomposed into four peaks (Figure S5a), while the C1s peak of GBC is decomposed into six peaks (Figure S5c). These six characteristic peaks of GBC can well match the five chemical components, including the C–C bond around 284.2 eV and 285.4 eV, C=C double bond around 284.8 eV, hydroxyl (C-OH) around 286.4 eV, carbonyl (C=O) around 287.7 eV, and carboxyl (-COOH) around 289.0 eV separately. This indicates that the intervention of pyromellitic dianhydride plays an important role in introducing carboxyl functional groups.

### 3.2. Influence of Initial pH Value

**Figure 1:**
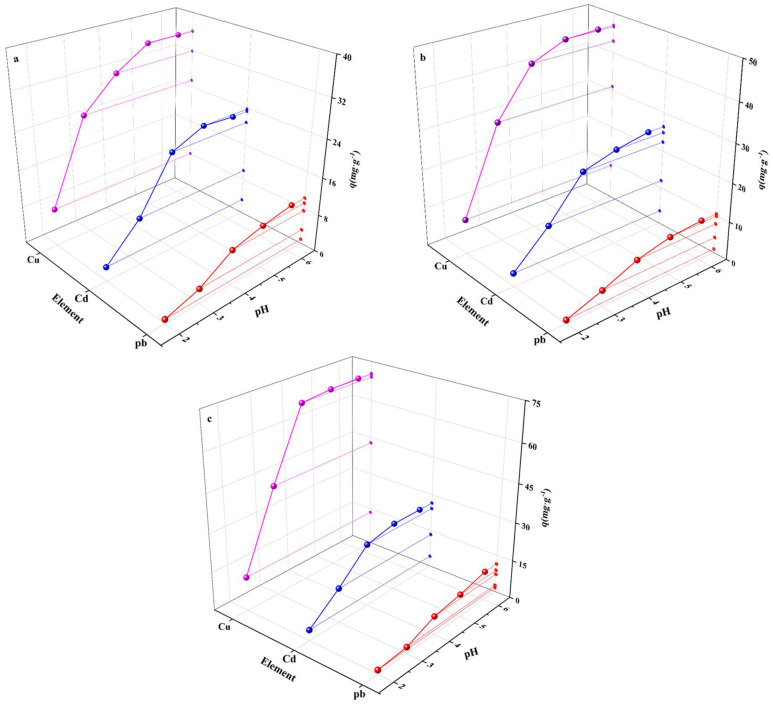
The effect of initial solution pH on the adsorption of heavy metals by BC (**a**), chitosan-modified biochar (**b**) and GBC (**c**).

The solution’s initial pH value has important implications on biochar adsorption (Figure 1). Figure 1 shows that when pH < 3, BC’s and GBC’s adsorption capacities for Pb, Cd, and Cu are very low; and increased pH means increased capacities. This is because H^+^ would preferentially bind to the functional groups on biochar’s surface in a low pH environment, which makes it difficult for heavy metal ions to bind. When the pH value gradually increases, the deprotonation effect could make heavy metal ions coordinate with the functional groups, thereby improving biochar’s adsorption capacity [36]. When the solution pH > 5, the adsorption capacities of BC and GBC for Cd, Pb, and Cu are stable with the increase in pH. This phenomenon matches the research results of Yao et al. [37]. They found that when the pH value goes up from 2 to 5, the adsorption amount of Cu also increased, and when the pH value > 5, the adsorption amount of Cu was stable. This phenomenon may be related to precipitation because it affects the adsorption of heavy metal ions [36].

### 3.3. Competitive Adsorption

Under competitive conditions (see Figure S6), the adsorption of Cu by GBC decreases by 13.75%, and the adsorption of Pb and Cd decreases by 48.18% and 68.03% separately. This indicates that compared with Cu, the effect of pH on Pb and Cd adsorption is more obvious in the polymetallic system.

Under different pH conditions, the K_d_ and α values of Pb, Cd, and Cu are in Table 1: except K_d_ (Pb), under the condition of Ph < 5, the values of Kd (Cu) and Kd (Cd) increase significantly with the increase in pH value; however, when pH > 5, the values of K_d_(Cu) and K_d_(Cd) do not increase significantly with the increase in pH. The increase in K_d_ value is due to the deprotonation effect. Increased pH would bring more activated adsorption sites and more metal ions to the biochar [38], thus being adsorbed by biochar. In addition, under different pH conditions, the selective adsorption of Cu and Cd by GBC is higher than that of Cu and Pb. According to the α value and the adsorption capacity of single metal on GBC, GBC shows a high affinity for Cu but strong adsorption inhibition for Pb and Cd in the competitive system. This means that the adsorption performance of GBC for Cu would be better than that of Cd and Pb.

### 3.4. Adsorption Kinetics

**Figure 2:**
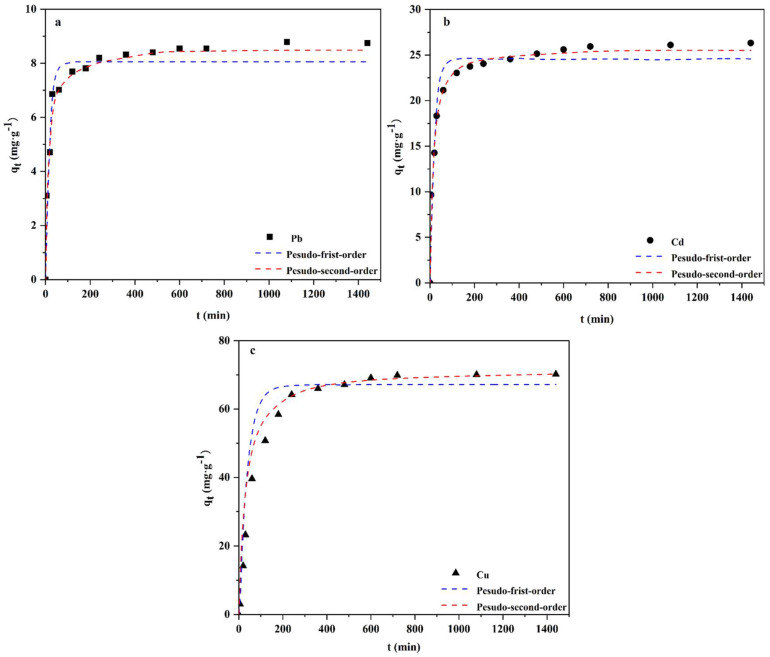
Kinetics of Pb (**a**), Cd (**b**), and Cu (**c**) after GBC adsorption.

The adsorption kinetics model is a common method to obtain the adsorption rate [39]. The effect of contact time on GBC adsorption is in Figure 2. It shows that in the first few hours, the adsorption rate is fast, but the adsorption rate declines with time, and it reaches equilibrium within 24 h. This result shows that the adsorption can be divided into two stages, namely, the stage of rapid migration of heavy metal ions from solution to biochar surface and the stage of slow diffusion from biochar surface to its internal. The adsorption capacities of Pb, Cd, and Cu at equilibrium are 8.81 mg·g^−1^, 26.48 mg·g^−1^, and 70.18 mg·g^−1^ separately.

The first stage of rapid migration of Pb, Cd, and Cu occurs within 1 h, 2 h, and 4 h, which may be related to the surface properties of GBC [40]. On GBC, more adsorption sites on Cu than Cd and Pb means that GBC can adsorb more Cu ions onto its surface in the first stage, so it takes the longest time.

Table 2 presents the results of adsorption kinetics. It shows that the fitting coefficients (R^2^) of the pseudo-first-order kinetic models (“the first model”) of Pb, Cd, and Cu are 0.9593, 0.9516, and 0.9788, respectively. The first model has a high fitting degree with the adsorption data only in the first two hours of the adsorption process (Figure 2). The fact that the data fitting degree of the whole experimental process is not high indicates that the first model cannot clarify the adsorption mechanism.

The equilibrium adsorption capacities in the first model are 8.23 mg·g^−1^, 24.64 mg·g^−1^, and 66.741 mg·g^−1^ separately. Fitting coefficients (R^2^) of the pseudo-second-order kinetic model (“the second model”) for Pb, Cd, and Cu are 0.9924, 0.9976, and 0.9971 separately. The fitting coefficients of the second model for Pb, Cd, and Cu are higher than those of the first model. This indicates kinetic data in the adsorption process aligns with the second model. In addition, the equilibrium adsorption capacities calculated with the model are 8.59 mg·g^−1^, 25.76 mg·g^−1^, and 71.42 mg·g^−1^ separately. These are closer to the experimental values. This result indicates that the adsorption process may involve electron exchange or chemical adsorption related to the dielectric gravity [41].

In Figure 2, compared with Cu, the time required for Pb and Cd to reach the adsorption equilibrium is relatively short. The K_2_ values of Pb, Cd, and Cu are 0.0117, 0.0034, and 0.0006 separately. This indicates the higher the K_2_ value is, the shorter the adsorption time is needed to reach equilibrium. The difference in K_2_ values among the three metals is related to the absorption rate, which is mainly determined by the surface properties of biochar [42].

In addition, we compared the maximum adsorption capacity of different adsorbents for heavy metals, and the results are shown in Table S3.

### 3.5. Adsorption Isotherms

**Figure 3:**
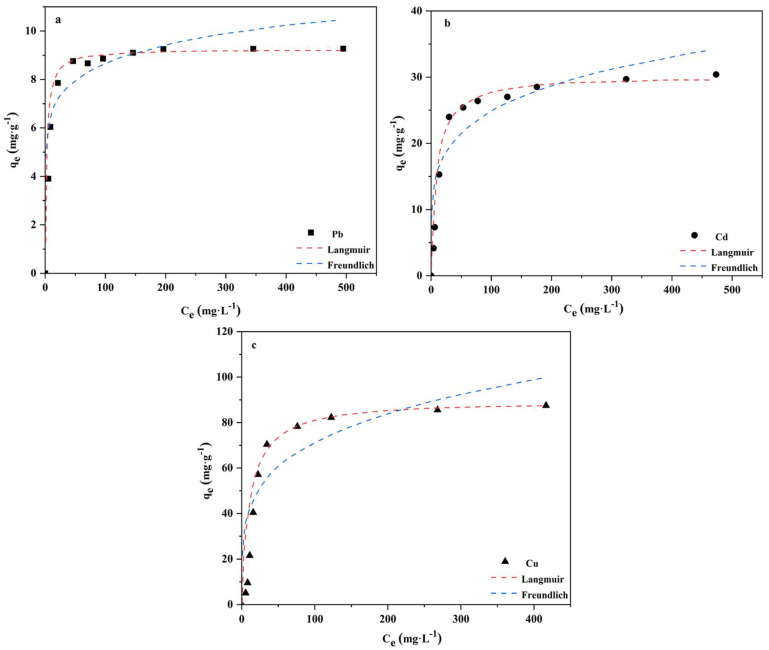
Isothermal lines of Pb (**a**), Cd (**b**), and Cu (**c**) after GBC adsorption.

The adsorption isotherms of Pb, Cd, and Cu by GBC are shown in Figure 3 and Table 3. They show that the fitting coefficients (R^2^) of the Freundlich model for Pb, Cd, and Cu are 0.9142, 0.8572, and 0.8599 separately, while the fitting coefficients (R^2^) of the Langmuir model for Pb, Cd, and Cu are 0.9348, 0.9912, and 0.9951. This indicates that the Langmuir model is more suitable than the Freundlich model to analyze the adsorption mechanism of GBC for Pb, Cd, and Cu. It also shows that monolayer adsorption is of significance in their removal.

In addition, the q_m_ values in Table 3 show that the affinity and adsorption capacity of GBC for Cu is higher than those of Cd and Pb.

### 3.6. Adsorption Mechanism

The mechanism of modified biochar on heavy metals usually involves complexation, ion exchange, physical adsorption, precipitation, and the combined effects of these actions [29]. The results of adsorption kinetics and isothermal experiments show that the removal is mainly dominated by the surface chemical adsorption of a single molecular layer. Usually, the functional groups on the biochar surface would affect the adsorption of heavy metal ions [43].

**Figure 4:**
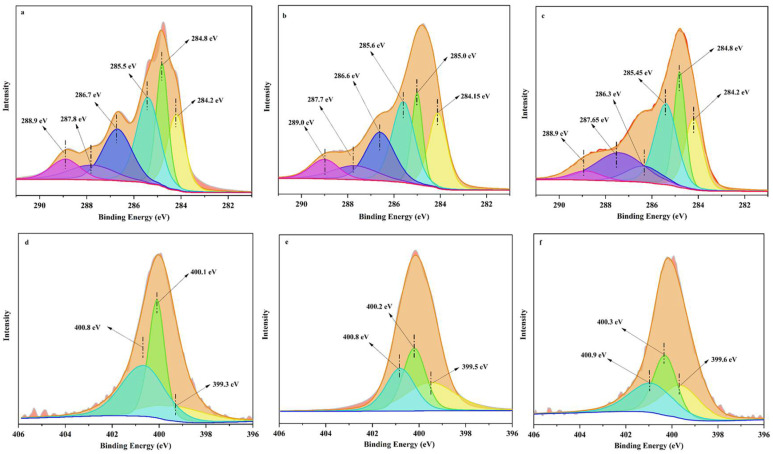
XPS Spectra of C1s and N1s after GBC adsorption. C1s: Pb (**a**), Cd (**b**) and Cu (**c**); N1s: Pb (**d**), Cd (**e**) and Cu (**f**).

The XPS peaks of C1s in GBC after adsorption of Pb, Cd, and Cu are shown in Figure 4a, Figure 4b, and Figure 4c, respectively. Compared with the XPS spectra of C1s in GBC before adsorption (Figure S5c), the characteristic peaks corresponding to the six types of carbon-containing functional groups do not change significantly after GBC adsorbed Pb (Figure 4a). This indicates that the removal effect of these six carbon-containing functional groups on Pb is not obvious. However, compared with the XPS spectrum of C1s element in GBC before adsorption, the characteristic peaks corresponding to the C=C double bond change significantly after GBC adsorbed Cd (Figure 4b), and the binding energy of the corresponding characteristic peaks also shifts significantly. This may be related to the complexation of the Л-electron, which forms the Cd–Л bond between the Cd and C=C double bond. It means that metal can form a σ bond with the s orbital of the C=C double bond, and the d orbital of metal can reversely contribute electrons to the Л orbital of the C=C double bond [44,45,46]. This result indicates that the C=C double bond may be vital to the adsorption of Cd. The C1s peak pattern after Cu adsorption by GBC (Figure 4c) is also different from that before Cu adsorption by GBC. The characteristic peaks corresponding to carbonyl, carboxyl, and hydroxyl groups change significantly, indicating that carbonyl, carboxyl, and hydroxyl are significant to Cu adsorption.

The XPS peaks of N1s elements in GBC after adsorption of Pb, Cd, and Cu are in Figure 4d, Figure 4e, and Figure 4f separately. Compared with the XPS spectra of N1s elements in GBC before adsorption (Figure S5d), the characteristic peaks corresponding to N-C=O change significantly after GBC adsorbs Pb (Figure 4d), and the binding energy of the corresponding characteristic peaks also shifts significantly. This result shows that N-C=O functional group is very important in the removal of Pb by GBC. After GBC adsorbs Cd (Figure 4e) and Cu (Figure 4f), the three characteristic peaks also change significantly. This suggests that N-containing functional groups participate in their adsorption.

In addition, after GBC adsorbs Cd and Cu, the characteristic peaks corresponding to these three N-containing functional groups also shift significantly. The obvious shift of these characteristic peaks may be due to complexes between N-containing functional groups and heavy metals. Studies have shown that when the covalent bond formed by N atoms and heavy metals obtains lone pair electrons from N atoms, the electron cloud density on N atoms decreases, thus changing the binding energy of the corresponding characteristic peaks [47] and shifting them.

Because the adsorption capacity of GBC for Cu is the largest and there are many functional groups involved in the Cu adsorption process in GBC, the Cu2p after GBC adsorption is further fitted by peak separation. In Figure S7, two characteristic peaks are in the peak plot of Cu2p, in which the characteristic peak of Cu^+^ is at 931 eV and the characteristic peak of Cu^2+^ is at 953 eV [48]. This indicates that the removal process of Cu^2+^ by GBC is accompanied by reduction, and the occurrence of Cu^2+^ reduction may be related to the existence of carbonyl in GBC. Studies have found that carbonyl groups can be used as electron donors for Cu^2+^ reduction to Cu^+^ [42].

Mineral components, such as K^+^, Ca^2+^, and carbonate in biochar, have some roles in the adsorption of heavy metal ions in an aqueous solution. Studies have shown that those components can ion exchange and precipitate with heavy metal [38], which will affect the removal effect of biochar. The adsorption of Pb on modified biochar only increases slightly, which may be related to the decrease in mineral components in GBC. XRD results of GBC show that KCl and CaCO_3_ disappeared after modification. Although the two mineral components of KCl and CaCO_3_ in modified GBC disappeared, the adsorption of Pb by GBC increases slightly. This may be because the complexing effect of the N-C=O functional group in GBC on Pb is greater than that of KCl and CaCO_3_ mineral components in GBC on Pb ion exchange and precipitation. The XPS results after Pb adsorption by GBC (Figure 4d) show that the characteristic peak of N-C=O changes significantly, indicating that N-C=O is vital to the process of Pb removal by GBC.

In summary, the chemical adsorption of a single molecular layer is the main adsorption mechanism of GBC on Pb, Cd, and Cu. In addition, the mineral components in GBC also have a certain contribution to its adsorption. In this study, the types of functional groups on biochar are closely related to the removal of heavy metals. For the adsorption of Pb, N-C=O functional groups are important, while other functional groups are less important. This may be the main reason that the removal capacity of GBC for Cd and Cu is higher.

This could be explained by: for Cd removal, there are three N-containing functional groups as well as C=C double bonds; and For Cu, GBC has the highest adsorption capacity, in addition to the participation of three N-containing functional groups, carboxyl groups, and hydroxyl groups, carbonyl functional groups are also involved in the reduction of Cu^2+^, which can reduce Cu^2+^ to Cu^+^ [42].

### 3.7. GBC Reproducibility and the Application of Actual Water Samples

The reusability and stability of adsorbent are of great importance for wastewater treatment. Good reusability and stability could help to reduce the operating costs of wastewater treatment. In this study, the renewable performance of GBC was investigated by five adsorption–desorption experiments using 0.1 mol·L^−1^ NaOH as a desorbent. The results are shown in Figure S8a. After five adsorption–desorption experiments, the adsorption performance of GBC decreased. The adsorption of Pb, Cd and Cu decreased from 9.33 mg·g^−1^, 30.14 mg·g^−1^ and 90.02 mg·g^−1^ in the first run to 8.65 mg·g^−1^, 26.89 mg·g^−1^ and 84.55 mg·g^−1^ in the fifth run, and the decline were 7.28%, 10.78% and 6.07% for Pb, Cd and Cu, respectively. After five adsorption–desorption experiments, the adsorption capacity of GBC for Pb, Cd and Cu did not decrease significantly, indicating that GBC has good renewable properties.

Considering that actual wastewater contains dissolved organic matter and co-existing ions, these dissolved organic matter and co-existing ions may compete for adsorption sites on GBC, thus affecting the adsorption of target pollutants by GBC. In this study, tap water and river water were used as the study subjects, and deionized water was used as a reference to assess the stability of GHC in practical applications. The parameters of different water samples were analyzed using pH meter, TOC analyzer, ICP-OES and UV–Vis spectrophotometer. The analysis results are shown in Table S4. As can be seen from Table S4, the organic matter and metal ion contents in tap water and river water were high. The adsorption results of GBC in different water samples are shown in Figure S8b. According to Figure S8b, the adsorption capacities of GBC for Pb, Cd and Cu in deionized water were 9.33 mg·g^−1^, 30.14 mg·g^−1^ and 90.02 mg·g^−1^, respectively. The adsorption capacities for Pb, Cd and Cu in tap water were mg·g^−1^, 29.88 mg·g^−1^ and 89.62 mg·g^−1^, respectively. The adsorption capacities for Pb, Cd and Cu in river water were 9.54 mg·g^−1^, 32.67 mg·g^−1^ and 93.47 mg·g^−1^, respectively. These results demonstrate the stable adsorption performance of GBC and also show that GBC has great potential for the removal of Cu from real wastewater.

## 4. Conclusions

In this study, a new type of biochar (GBC) was successfully prepared by using silkworm sand as raw material to prepare biochar (BC), which was then modified with chitosan combined with pyromellitic dianhydride. GBC is then used to adsorb Cd, Pb, and Cu in an aqueous solution, and the adsorption mechanism is discussed. Compared with the original biochar (BC), the removal of Pb and Cd by GBC was increased by approximately 12% and that of Cu by approximately 94.6%, respectively. In addition, in the polymetallic system, GBC has the strongest selective adsorption of Cu. The pseudo-second-order kinetics model and the Langmuir model can well describe the kinetics and isotherms of GBC. This indicates that the chemical adsorption of the single-molecule layer is vital to the adsorption of Pb, Cd, and Cu. It also shows the types of functional groups in biochar have a great impact on their adsorption. Five adsorption–desorption experiments on GBC showed that the reduction in adsorption of Pb, Cd and Cu was 7.28%, 10.78% and 6.07%, respectively, indicating that GBC has good regenerative properties. In addition, the adsorption amounts of Cu in different water samples ranged from 89.62 to 93.47 mg·g^−1^, indicating that GBC has great potential for the removal of Cu from wastewater.

## Figures and Tables

**Table 1:** Distribution coefficient and selectivity coefficient of competitive adsorption of Pb, Cd, and Cu on GBC.

pH	K_d_(Pb)	K_d_(Cd)	K_d_(Cu)	$\alpha_{Cd}^{Cu}$	$\alpha_{Pb}^{Cu}$
2	0.0071	0.0133	0.1026	7.6232	13.8061
3	0.0137	0.0456	0.6563	14.2818	48.0182
4	0.0326	0.0721	1.4246	21.8139	43.4567
5	0.0503	0.1028	1.7428	16.8621	34.4987
6	0.0536	0.0994	1.7616	17.9672	33.6263

**Table 2:** Pseudo-first-order kinetic model and pseudo-second-order kinetic model parameters of Pb, Cd, and Cu after GBC adsorption.

Element	Pseudo-First-Order			Pseudo-Second-Order		
	K_1_ (min^−1^)	q_e_ (mg·g^−1^)	R^2^	K_2_ (g·(mg·min)^−1^)	q_e_ (mg·g^−1^)	R^2^
Pb	0.0631	8.2339	0.9593	0.0117	8.5933	0.9924
Cd	0.0558	24.6435	0.9516	0.0034	25.7601	0.9976
Cu	0.0269	66.7413	0.9788	0.0006	71.4227	0.9971

**Table 3:** Parameters of Freundlich and Langmuir isothermal models for Pb, Cd and Cu after GBC adsorption.

Element	Freundlich			Langmuir		
	K_F_	1/n	R^2^	K_L_	q_m_	R^2^
Pb	5.1107	0.1193	0.9142	0.5339	9.33	0.9348
Cd	9.8432	0.2108	0.8572	0.1197	30.14	0.9912
Cu	23.6736	0.2419	0.8599	0.0949	90.02	0.9951

## Data Availability

Not applicable.

## Supplementary Materials

The following supporting information can be downloaded at: https://www.mdpi.com/article/10.3390/polym14142889/s1: Detailed description of adsorption experiments, Figure S1: SEM images of BC (a) and GBC (b), Figure S2: XRD patterns of BC and GBC, Figure S3: FTIR spectra of BC and GBC, Figure S4: XPS full-spectrum scanning diagram of BC and GBC, Figure S5: XPS Spectra of C1s (a), N1s (b) in BC, and C1s (c), N1s (d) in GBC, Figure S6: Effect of initial pH value of the solution on competitive adsorption of Cd, Pb, and Cu on GBC, Figure S7: XPS peak plot of Cu2p in GBC after Cu adsorption, Figure S8: The adsorption-desorption cycles of Pb, Cd and Cu on GBC (a); adsorption of Pb, Cd and Cu by GBC in different water samples (b), Table S1: BC and GBC surface properties and ash content, Table S1: BC and GBC surface properties and ash content, Table S2: Content of C, N, and O elements in BC and GBC (%), Table S3: Comparison of maximum adsorption capacity of heavy metal with various adsorbents, Table S4: Water sample parameters of deionized water, tap water and river water.
