# Supplementary material for: Removal and Mechanism of Cadmium, Lead and Copper in Water by Functional Modification of Silkworm Excrement Biochar
Source: Polymers (Basel). 2022 Jul 16;14(14):2889. doi: 10.3390/polym14142889 (PMC9323519; doi:10.3390/polym14142889)
Supplement: Supplementary file 1 [file polymers-14-02889-s001.zip › polymers-1801008-supplementary.pdf]

Removal and mechanism of cadmium, lead and copper in water by functional modification of silkworm excrement biochar

Pengyang Bian [1], Yixuan Liu [1], Xiaoqin Zheng [2] and Weibo Shen [1,3,*]

[1] College of Natural Resources and Environment, Northwest A&F University, Xianyang 712100, China; bianpy@nwafu.edu.cn(P.B.); liuyx20@nwafu.edu.cn (Y.L.)

[2] Qinba Ecological Protection Center, Hanzhong 723000, China; zkyjys123@126.com

[3] Institute of Soil and Water Conservation, Northwest A&F University, Xianyang 712100, China

* Correspondence: shenweibo@nwafu.edu.cn; Tel./Fax: +8629-87019629

Detailed de`scription of adsorption experiments

$Pb(HNO_3)_2$, ($Cu(NO_3)_2·3H_2O$), and ($Cd(NO_3)_2·4H_2O$) were made into 1.0 g·L^{-1} Pb, Cu, and Cd standard reserve solution by deionized water.

pH experiment: by adjusting the original pH value of the solution to explore its effect on the adsorption performance of biochar: take 100 mg·L^{-1} solution containing Pb, Cd, and Cu respectively and place them in a group of 100 mL centrifuge tubes. Adjusting pH of Pb, Cu, and Cd to 2-7 by HNO_3 and NaOH respectively. Then a 1.0 g·L^{-1} GBC sample was added to Pb, Cd, and Cu solutions respectively. Then, it oscillated at 220 r·min^{-1} for 24 h in a 25±1℃ thermostatic oscillator. After centrifugation at 4000 r·min^{-1} for 10 mins, the supernatant was filtered by 0.45 μm microporous membranes (GE cellulose nylon membrane). The filtered solution containing heavy metals was determined on an Inductively Coupled Plasma Mass Spectrometer (ICP-MS, NexION 2000, Perkin-Elmer SCIEX, USA). Three replicate samples with BC were set as control groups. The concentration difference between the initial solution and the final solution was used to calculate the amount of heavy metal that biochar adsorbed. The formula is as follows:

$$q_e = \frac{(C_0 - C_e) \times V}{m} \tag{1}$$

C_0 (mg·L^{-1}) and C_e (mg·L^{-1}) represent the initial concentration and equilibrium concentration of heavy metals respectively. V(L) is the solution volume, and m (g) is the mass of the adsorbent.

Adsorption kinetics: we took 50mL of 100 mg·L^{-1} solutions containing Pb, Cd, and Cu respectively, and placed them in a group of 100 mL centrifuge tubes. Then we adjusted pH to 5, then added 1.0 g·L^{-1} GBC. Then the centrifuge tube was placed in a constant temperature oscillation machine at 25±1℃ to oscillate at 220 r·min^{-1} and sampled at different time points (5-1440 mins). The concentration of residual heavy metals in the solution was determined. The centrifugation, filtration, and determination of the heavy metal concentration of the solution were the same as those in pH experiment in (1). Three repetitions are set for each processing. Pseudo-first-order kinetic model (formula 2) and pseudo-second-order kinetic model (formula 3) were used to fit the experimental data [1]. The formula is as follows:

$$\ln(q_e - q_t) = \ln q_e - k_1 t \tag{2}$$

$$\frac{t}{q_t} = \frac{1}{k_2 q_e^2} + \frac{t}{q_e} \quad (3)$$

In the formula, q_t and q_e(mg·g^{-1}) refer to the adsorption capacities at t moment and the adsorption equilibrium, and K_1和K_2 refers to the rate constants of the pseudo-first-order kinetic model and the pseudo-second-order kinetic model.

Adsorption isotherm: we adjusted the pH of solutions respectively containing different concentrations of Pb, Cd, and Cu (0.5-500 mg·L^{-1}) to 5. Then we took 50 mL of Pb, Cd, and Cu solutions and put them into a group of 100 mL centrifuge tubes respectively, and 1.0 g·L^{-1} of GBC was added to each of them. Then, the centrifuge tube was placed in a 25±1℃ thermostatic shaker and oscillated at 220 r·min^{-1} for 24 h for sampling. The concentration of residual heavy metals in the solution was determined. The centrifugation, filtration, and determination of the heavy metal concentration of the solution were the same as those in pH experiment in (1). Three repetitions are set for each processing. Freundlich (formula 4) and Langmuir (formula 5) isotherm models were applied to fit the analysis data [2, 3]. The formula is as follows:

$$q_{e=}K_F\, C_e^{1/n} \quad (4)$$

$$q_e = \frac{C_e q_m K_L}{1 + C_e K_L} \quad (5)$$

In the equation, K_F refers to the adsorption constant of the Freundlich isothermal model, and 1/n refers to the surface heterogeneity or adsorption strength: when 1/n < 1, it is the normal Freundlich isotherm; and when 1/n > 1, it is synergistic adsorption (Tan et al., 2015a). C_e represents the concentration of heavy metal ions at adsorption equilibrium (mg·L^{-1}). q_m is the maximum adsorption capacity of GCB (mg·g^{-1}). K_L represents the adsorption constant of the Langmuir isotherm model. C_0 represents the initial concentration of the heavy metal solution (mg·L^{-1}).

Competitive adsorption: the mixed solution containing Pb, Cd, and Cu was prepared, and the concentrations of Pb, Cd, and Cu in the mixed solution were 100 mg·L^{-1}. Then 50mL mixed solution containing Pb, Cd, and Cu was added to a group of 100mL centrifuge tubes, and the pH was adjusted to 2-6. Then, the centrifuge tube containing the mixture was placed in a 25±1℃ thermostatic shaker and oscillated at 220 r·min^{-1} for 24 h for sampling. The concentration of residual heavy metals in the solution was determined. The centrifugation, filtration, and determination of heavy metal ion concentration of the solution were the same as those in the

pH experiment in (1). Three repetitions are set for each processing. The distribution coefficient (Kd) was used to analyze the selective adsorption of Pb, Cd, and Cu by GCB [4], and the formula is as follows:

$$K_d = \frac{V \times (C_0 - C_e)}{mC_e} \quad (6)$$

In the formula, C_0 ($mg \cdot L^{-1}$) and C_e ($mg \cdot L^{-1}$) represent the initial concentration and equilibrium concentration of heavy metal ions. Moreover, V (L) is the solution volume, and m (g) is the mass of the adsorbent.

In addition, in the presence of interfering ions, the selectivity coefficient (α) of specific adsorbates is calculated by the following formula:

$$\alpha = \frac{K_d(T)}{K_d(I)} \quad (7)$$

In this study, K_d (T) represents the K_d value in Cu and K_d (I) represents the K_d value Pb and Cd. The greater the α value, the stronger the selective adsorption capacity of Cu is than that of Pb and Cd.

Figure captions

Figure S1. SEM images of BC (a) and GBC (b).

Figure S2. XRD patterns of BC and GBC.

Figure S3. FTIR spectra of BC and GBC.

Figure S4. XPS full-spectrum scanning diagram of BC and GBC.

Figure S5. XPS Spectra of C1s (a), N1s (b) in BC, and C1s (c), N1s (d) in GBC.

Figure S6. Effect of initial pH value of the solution on competitive adsorption of Cd, Pb, and Cu on GBC.

Figure S7. XPS peak plot of Cu2p in GBC after Cu adsorption.

Figure S8. The adsorption-desorption cycles of Pb, Cd and Cu on GBC (a); adsorption of Pb, Cd and Cu by GBC in different water samples (b).

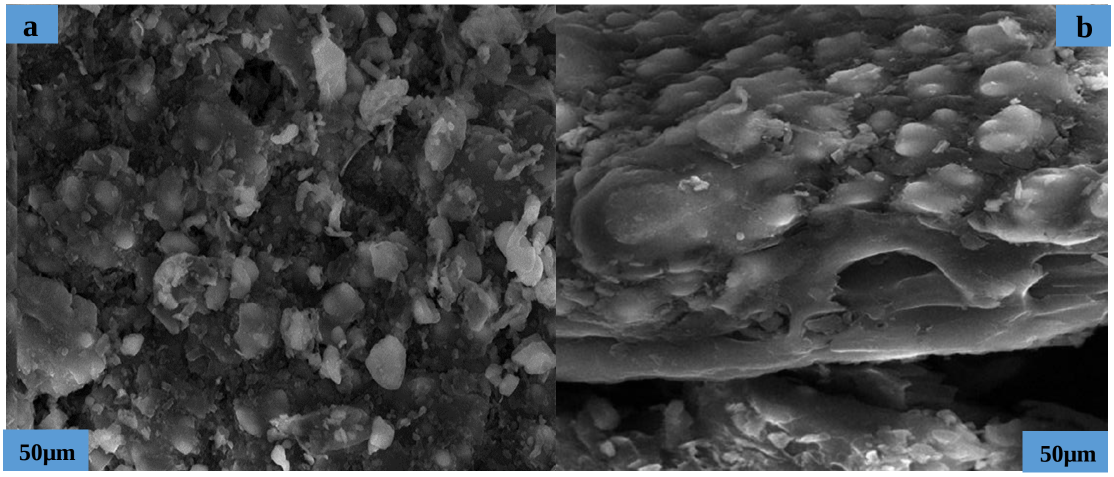

Figure S1. SEM images of BC (a) and GBC (b).

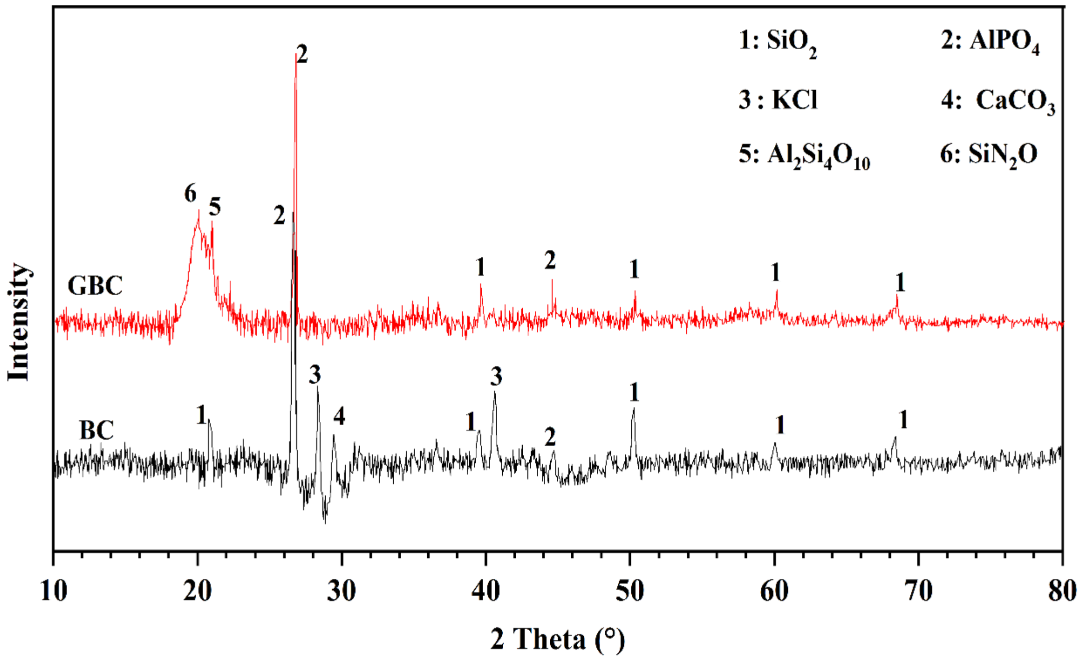

Figure S2. XRD patterns of BC and GBC.

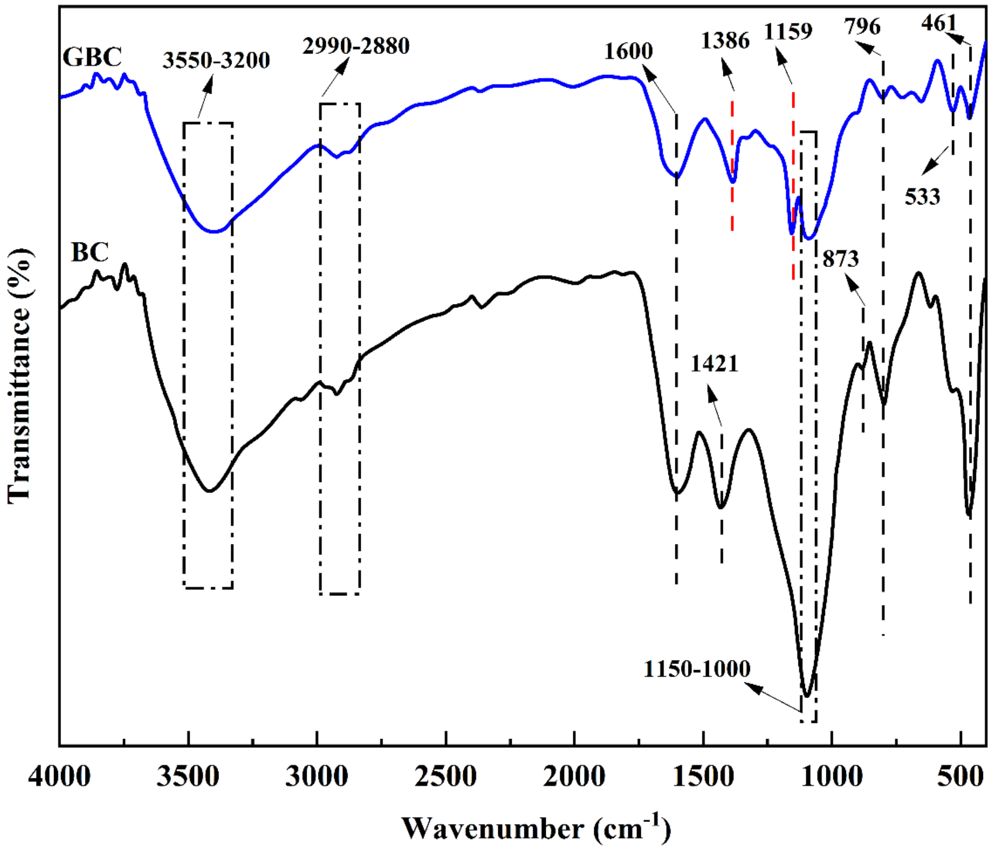

Figure S3. FTIR spectra of BC and GBC.

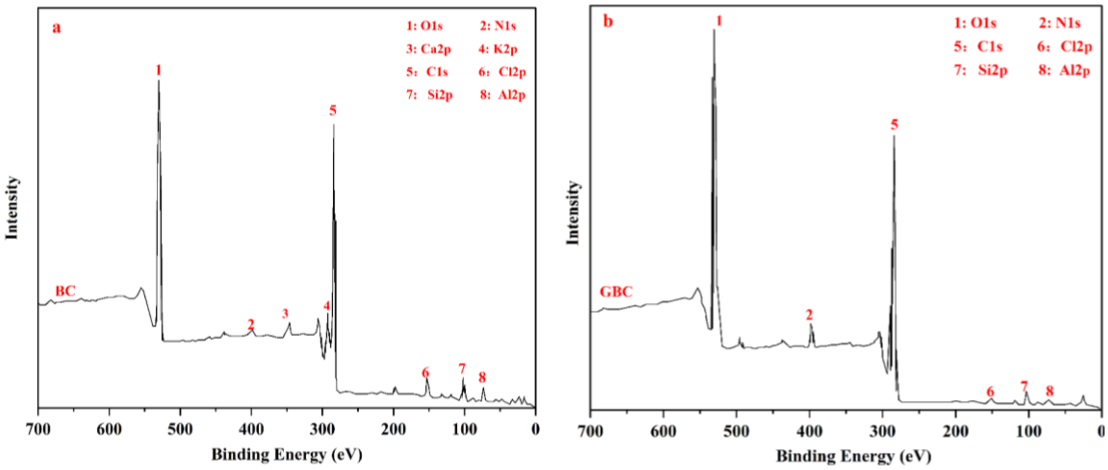

Figure S4. XPS full-spectrum scanning diagram of BC and GBC.

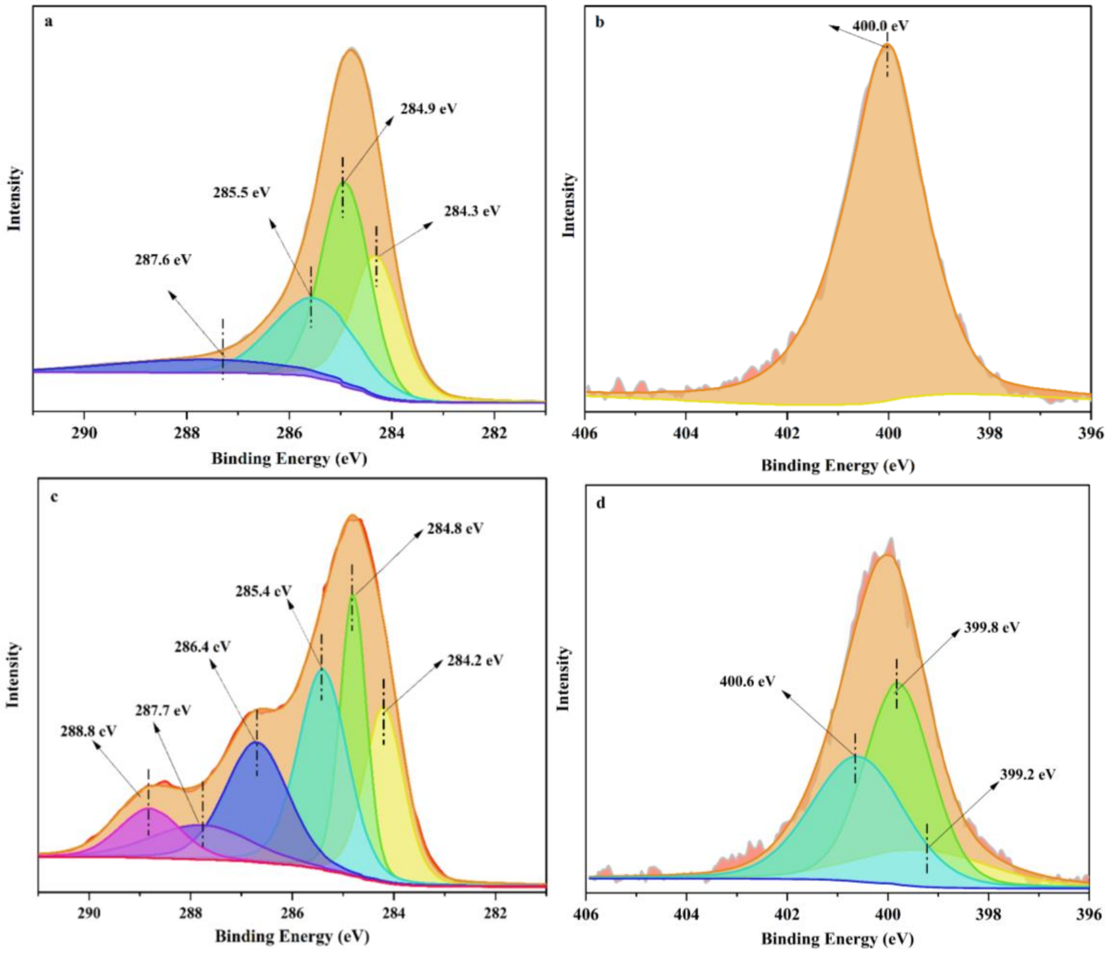

Figure S5. XPS Spectra of C1s (a), N1s (b) in BC, and C1s (c), N1s (d) in GBC.

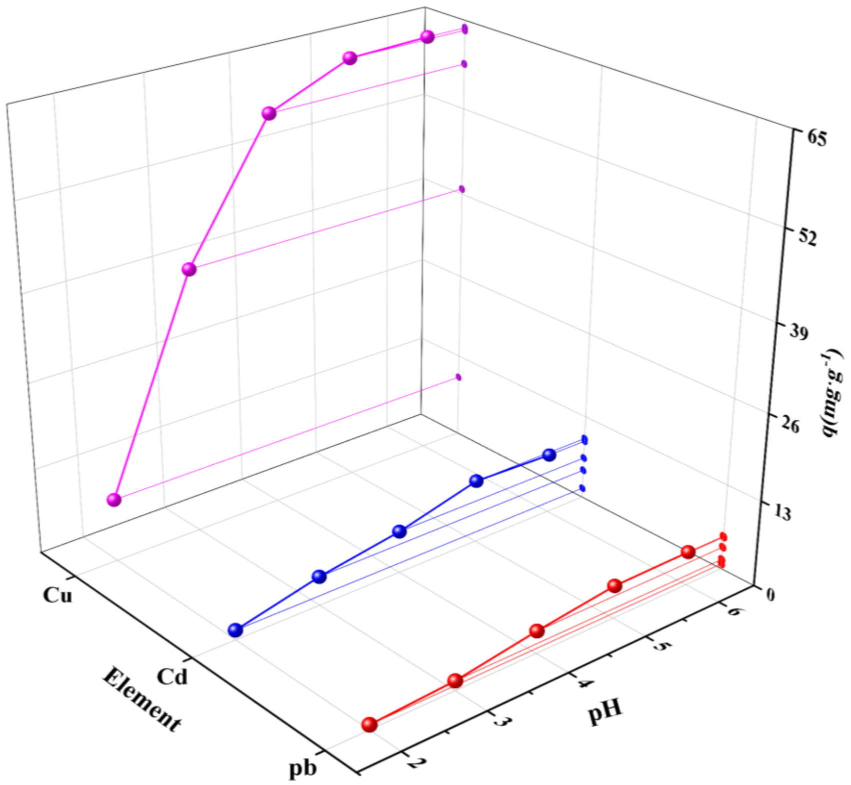

Figure S6. Effect of initial pH value of the solution on competitive adsorption of Cd, Pb, and Cu on GBC.

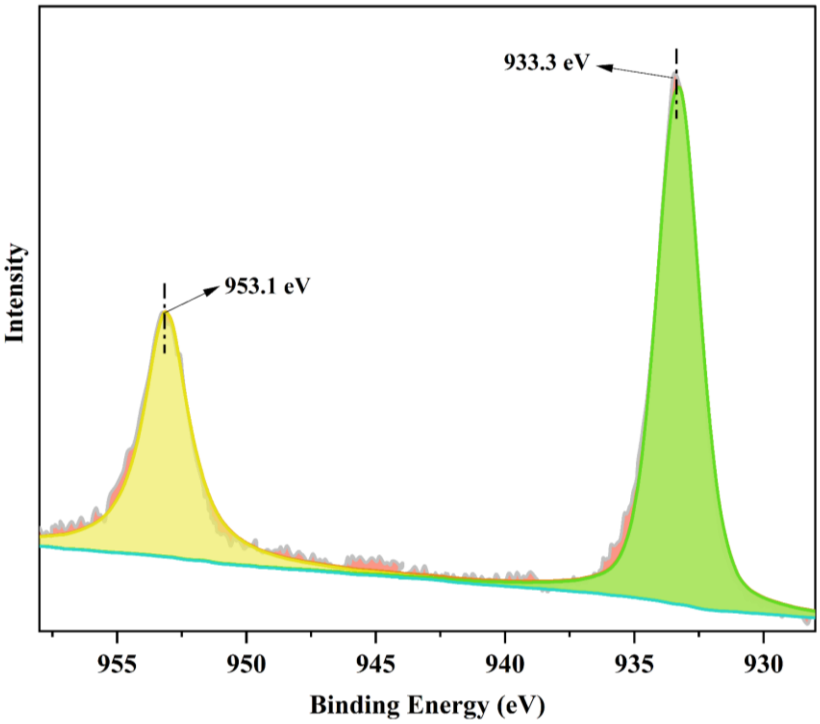

Figure S7. XPS peak plot of Cu2p in GBC after Cu adsorption.

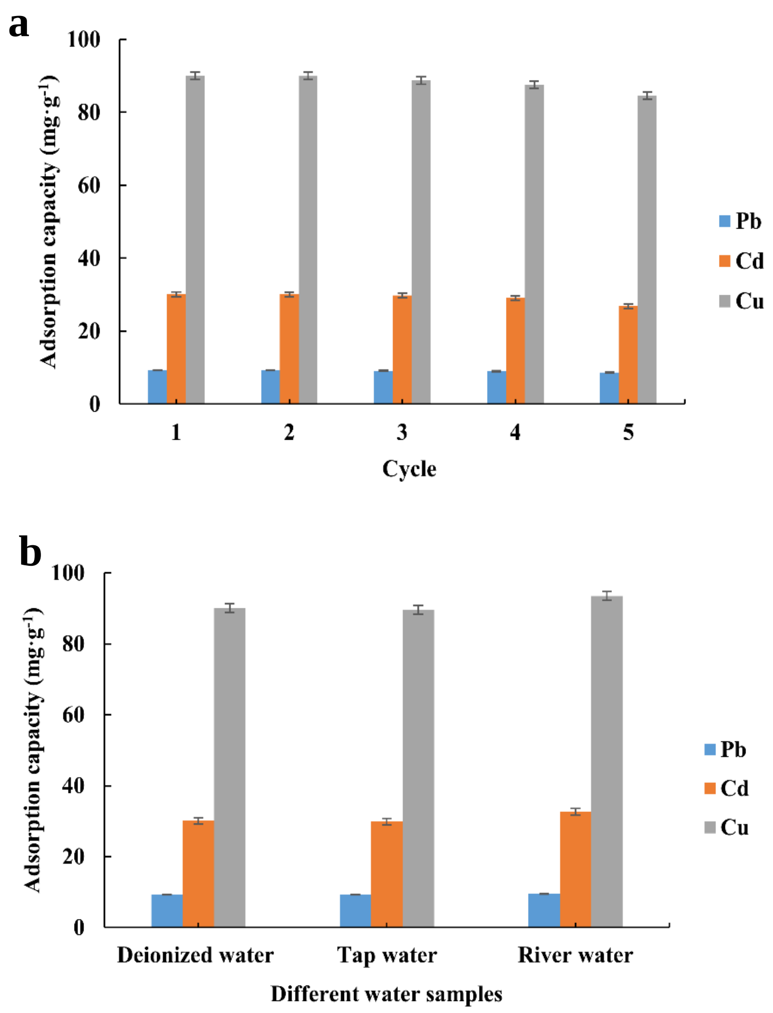

Figure S8. The adsorption-desorption cycles of Pb, Cd and Cu on GBC (a); adsorption of Pb, Cd and Cu by GBC in different water samples (b).

Table captions

Table S1. BC and GBC surface properties and ash content.

Table S2. Content of C, N, and O elements in BC and GBC (%).

Table S3. Comparison of maximum adsorption capacity of heavy metal with various adsorbents.

Table S4. Water sample parameters of deionized water, tap water and river water.

Table S1. BC and GBC surface properties and ash content.

Material	surface area	Pore volume	Ash
	$m^2 \cdot g^{-1}$	$cm^3 \cdot g^{-1}$	%
BC	28.4	0.031	29.6
GBC	68.2	0.087	21.8

Table S2. Content of C, N, and O elements in BC and GBC (%).

**Materials**	**Atomic %**		
	**C**	**N**	**O**
BC	73.81	2.31	22.63
GBC	61.23	9.27	28.04

Table S3 Comparison of maximum adsorption capacity of heavy metal with various adsorbents.

Adsorbents	Temperature (℃)	Q_{max}(mg·g^{-1}) Pb	Cd	Cu	Reference
GBC	25	9.33	30.14	90.02	This study
AC	25			13.81	Thuan et al. [5]
GGAC	25	20.3	27.3		Asuquo et al. [6]
AC	25			37.17	Demiral et al. [7]
OAC	25		13.38		Polo and Utrilla [8]
SABC	25			43.88	Li et al. [9]
CAC	25		35.71	33.33	Kannan and Veemaraj [10]
P1	25	27.39	4.43		Zhang et al. [11]
P6	25	67.45	12.93		Zhang et al. [11]

Table S4. Water sample parameters of deionized water, tap water and river water.

Water sample	pH	TOC	Element content (mg·L^{-1})				
		(mg·L^{-1})	K	Na	Ca	Mg	P
Deionized water	6.8	0	0	0	0.003	0	0.001
Tap water	7.1	0.37	4.68	10.69	34.22	5.64	0.01
River water	7.3	5.28	6.09	11.79	33.32	5.76	0.05

Reference

1. Yang, F.; Zhang, S.; Sun, Y.; Tsang, D. C. W.; Cheng, K.; Ok, Y. S., Assembling biochar with various layered double hydroxides for enhancement of phosphorus recovery. *Journal of Hazardous Materials* **2019,** 365, 665-673.
2. Prasad, M.; Xu, H.-y.; Saxena, S., Multi-component sorption of Pb(II), Cu(II) and Zn(II) onto low-cost mineral adsorbent. *Journal of Hazardous Materials* **2008,** 154, (1), 221-229.
3. Tan, X.; Liu, Y.; Gu, Y.; Zeng, G.; Wang, X.; Hu, X.; Sun, Z.; Yang, Z., Immobilization of Cd(II) in acid soil amended with different biochars with a long term of incubation. *Environmental Science and Pollution Research* **2015,** 22, (16), 12597-12604.
4. Li, K.; Wang, Y.; Huang, M.; Yan, H.; Yang, H.; Xiao, S.; Li, A., Preparation of chitosan-graft-polyacrylamide magnetic composite microspheres for enhanced selective removal of mercury ions from water. *Journal of Colloid and Interface Science* **2015,** 455, 261-270.
5. Van Thuan, T.; Quynh, B. T. P.; Nguyen, T. D.; Ho, V. T. T.; Bach, L. G., Response surface methodology approach for optimization of Cu^{2+}, Ni^{2+} and Pb^{2+} adsorption using KOH-activated carbon from banana peel. *Surfaces and Interfaces* **2017,** 6, 209-217.
6. Asuquo, E.; Martin, A.; Nzerem, P.; Siperstein, F.; Fan, X., Adsorption of Cd(II) and Pb(II) ions from aqueous solutions using mesoporous activated carbon adsorbent: Equilibrium, kinetics and characterisation studies. *Journal of Environmental Chemical Engineering* **2017,** 5, (1), 679-698.
7. Demiral, H.; Güngör, C., Adsorption of copper(II) from aqueous solutions on activated carbon prepared from grape bagasse. *Journal of Cleaner Production* **2016,** 124, 103-113.
8. Sánchez-Polo, M.; Rivera-Utrilla, J., Adsorbent−Adsorbate Interactions in the Adsorption of Cd(II) and Hg(II) on Ozonized Activated Carbons. *Environmental Science & Technology* **2002,** 36, (17), 3850-3854.
9. Li, M.; Liu, Q.; Guo, L.; Zhang, Y.; Lou, Z.; Wang, Y.; Qian, G., Cu(II) removal from aqueous solution by Spartina alterniflora derived biochar. *Bioresource Technology* **2013,** 141, 83-88.
10. Momčilović, M.; Purenović, M.; Bojić, A.; Zarubica, A.; Ranđelović, M., Removal of lead(II) ions from aqueous solutions by adsorption onto pine cone activated carbon. *Desalination* **2011,** 276, (1), 53-59.
11. Zhang, C.; Shan, B.; Tang, W.; Zhu, Y., Comparison of cadmium and lead sorption by Phyllostachys pubescens biochar produced under a low-oxygen pyrolysis atmosphere. *Bioresource Technology* **2017,** 238, 352-360.
